# Supplementary material for: Cold Response of the Mediterranean Fruit Fly (Ceratitis capitata) on a Lab Diet
Source: Insects. 2019 Feb 3;10(2):48. doi: 10.3390/insects10020048 (PMC6409936; doi:10.3390/insects10020048)
Supplement: Supplementary file 1 [file insects-10-00048-s001.pdf]

Table S1: Four different models were used in the *C. capitate* mortality data analysis

		**Pupation method**								**Adult method**					
Name	Log	Type	Residue	BIC	Stage	Intercept	Slope	Name	Log	Type	Residue	BIC	Stage	Intercept	Slope
Model1	Y	Probit	209.9	345.48	Early eggs	-0.6943	1.52913	Model1	Y	Probit	220	347.11	Early eggs	-0.5642	1.53
Model2	Y	Logit	271.85	407.43		-1.235	2.729	Model2	Y	Logit	271.14	398.24		-1.011	2.758
Model3	N	Probit	147.12	282.7		-1.0024	0.5802	Model3	N	Probit	191.82	318.92		-0.915	0.5987
Model4	N	Logit	152.68	288.25		-1.819	1.049	Model4	N	Logit	188.1	315.21		-1.711	1.104
Model1	Y	Probit	210.73	342.4	Late eggs	-0.2423	1.3071	Model1	Y	Probit	231.9	358.6	Late eggs	0.1045	1.123
Model2	Y	Logit	288.82	420.49		-0.4303	2.331	Model2	Y	Logit	302.54	429.24		0.1641	2.0417
Model3	N	Probit	66.96	198.63		-0.5987	0.5247	Model3	N	Probit	82.99	209.69		-0.2035	0.4442
Model4	N	Logit	90.87	222.54		-1.1329	0.9518	Model4	N	Logit	111.05	237.75		-0.4842	0.8267
Model1	Y	Probit	492.6	664.82	1st instar	-2.225	2.063	Model1	Y	Probit	375.54	530.17	1st instar	-1.179	1.704
Model2	Y	Logit	445.94	618.15		-4.48	4.01	Model2	Y	Logit	431.46	586.1		-2.194	3.083
Model3	N	Probit	351.57	523.79		-2.0967	0.6199	Model3	N	Probit	259.42	414.05		-1.427	0.6155
Model4	N	Logit	298.65	470.87		-3.87	1.155	Model4	N	Logit	242.01	396.65		-2.6	1.123
Model1	Y	Probit	165.59	282.7	2nd instar	-3.097	2.991	Model1	Y	Probit	214.95	341.94	2nd instar	-1.718	2.178
Model2	Y	Logit	164.01	281.11		-5.903	5.616	Model2	Y	Logit	241.16	368.14		-3.317	4.068
Model3	N	Probit	98.43	215.54		-3.089	1.024	Model3	N	Probit	100.8	227.79		-2.1792	0.8784
Model4	N	Logit	92.74	209.85		-5.601	1.86	Model4	N	Logit	100.45	227.43		-3.873	1.566
Model1	Y	Probit	615.83	761.53	3rd instar	-2.755	2.292	Model1	Y	Probit	363.74	496.24	3rd instar	-1.522	1.94
Model2	Y	Logit	666.71	812.41		-5.038	4.133	Model2	Y	Logit	422.09	554.6		-2.801	3.503
Model3	N	Probit	465.81	611.51		-2.354	0.636	Model3	N	Probit	282.62	415.13		-1.8138	0.7244
Model4	N	Logit	504.37	650.07		-4.069	1.102	Model4	N	Logit	260.63	393.14		-3.125	1.258

The models including probit, logit, non log - probit and non - log logit. Which its blow a line was the selected one to calculate LT90 and LT99 in table1.

Table **S2: The sex ratios comparative of *C. capitata* stages which were treated with cold treatment 0.0 °C.**

SUMMARY

Groups	*Count*	*Sum*	*Average*	*Variance*
Early egg	15	776.6	51.7	98.1
Late egg	12	585.8	48.8	148.8
1st instar	18	939.7	52.2	65.6
2nd instar	14	726.2	51.8	89.8
3rd instar	18	872.6	48.4	109.5

ANOVA

Source of Variation	*SS*	*df*	*MS*	*F*	*P-value*	*F crit.*
Between Groups	208.38	4	52.09	0.52	0.71	2.49
Within Groups	7159.87	72	99.44			
Total	7368.25	76				

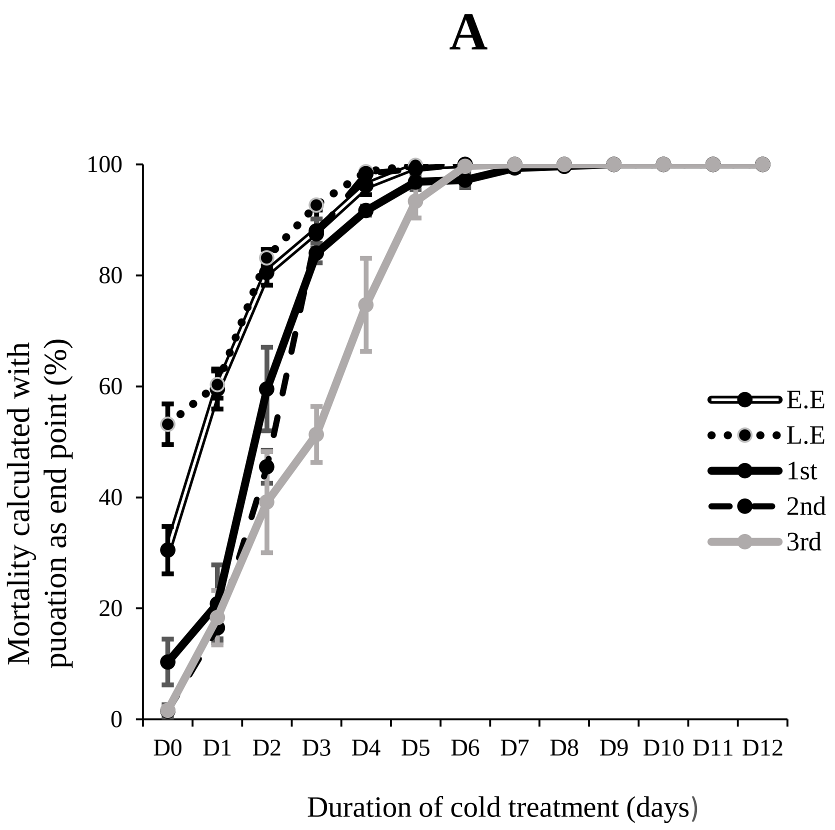

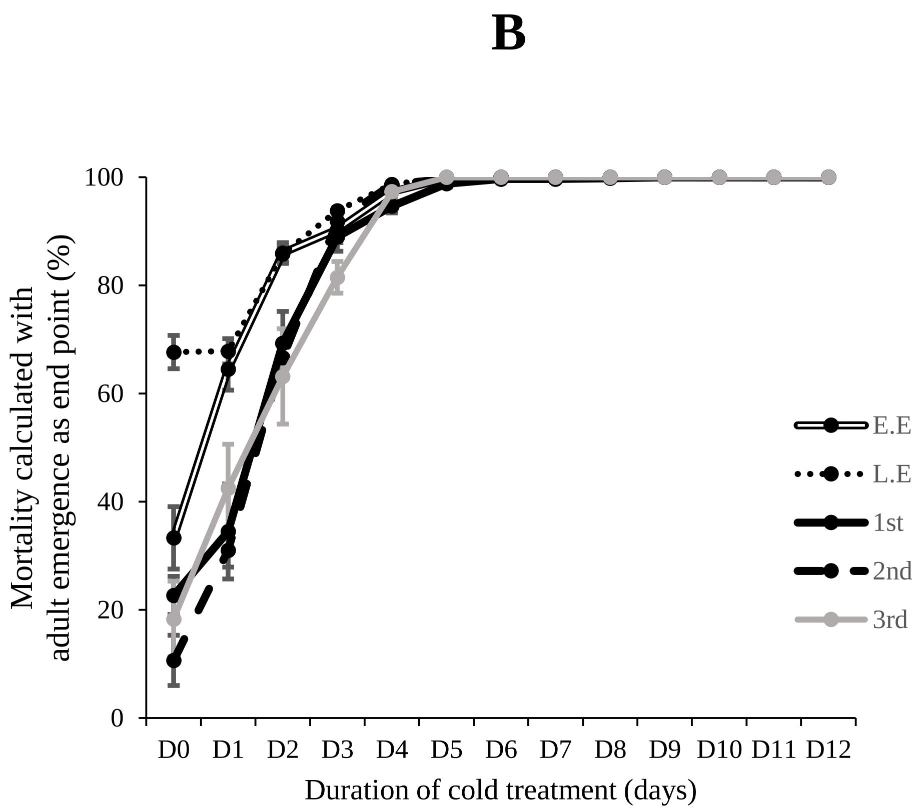

S1 Fig. (A) Mortality (%) based on pupation and adults (B) ratios from the treated *C. capitata*
